# Bioplasm

**Published:** 1872-10-15

**Authors:** D. T. Nelson

**Affiliations:** Professor of Physiology and Histology


### BIOPLASM.

An Address, Introductory to the Regular Course of Lectures in Chicago Medical College, Session of 1872—73.

BY D. T. NELSON, M. D.,

PROFESSOR OF PHYSIOLOGY AND HISTOLOGY.

*Ladies, and Gentlemen of the Entering Class:* —

The duty of welcoming you to these halls of learning, has been assigned to me by my colleagues. And it is a pleasant task, as we take you by the hand, to point you to some of the grand thoughts which must occupy your attention here.

But in passing, I must remind you that ours is a fast age, a fast nation, and a fast city. We go journeys in days our fathers travelled only in months. We acquire fortunes in years, which have required generations to amass in other countries.

We live in a city, which, less than a year ago, was almost without a business-house, and 100,000 of our citizens were without homes. To-day, we are told, there are more square feet of business-floors, and 50,000 more people sheltered in Chicago than before the Great Fire. And we have not yet celebrated its first anniversary. With such intense energy around you, you must be stimulated to unwonted activity. And if this shall be in the direction of faithful, thorough, and continued study of the topics of medical science as presented to you here, we feel confident that you will be satisfied with your progress, when the end of the session shall have been reached.

But if any one of you is so deceived as to suppose that medical science is easy — that all that will be needed in practice can be acquired in a few weeks, and with little labor — we would tell such an one, candidly, at the outset, you have mistaken your calling, you are in the wrong place, and we recommend you to seek some other field of labor. The physician must be a student always! His work is never done! But it was not my purpose to consider to any length the peculiarities of the life of a physician, or of the life of our age, or of our nation, or of our city, nor, indeed, of the human being, as distinguished from other living organisms. This shall be our study later in the term.

But we ask your attention now, simply to questions concerning living matter, or Bioplasm. Its origin. Its multiplication or growth. Its function. Questions which some of the best minds of the scientific world have been hard at work upon for years, and especially recently.

Living matter has of late received a variety of names, and before we proceed, we should fix upon the name which shall best embody the idea we entertain of it. We find, among recent writers, the terms, Germinal Matter, Protoplasm, Sarcode, and Bioplasm, and among the older writers, Aristotle speaks of animating principles. Heraclitus, of a universal fire. Hunter of a materia vitæ diffusa. Hoffman of a vital fluid, and Humboldt of a vital force.

All of these older writers evidently wished to convey to our minds the idea that living matter possessed a force, an energy — a something, peculiar to itself.

Of the recent terms, Protoplasm is undoubtedly the most familiar — made so by the writings of Prof. Huxley and his reviewers. We throw aside this term, more from the ill-repute and indefiniteness it has acquired in this spirited contest, than from any intrinsic defect in the term itself. As an example, let me give you a few of Prof .Huxley's words: "What community of faculty can there be between the brightly colored lichen, which so nearly resembles a mere mineral incrustation of the bare rock on which it grows, and the painter, to whom it is instinct with beauty, or the botanist, whom it feeds with knowledge? Again, think of the microscopic fungus — a mere infinitesimal ovoid particle — which finds space and duration enough to multiply into countless millions in the body of a living fly; and then of the wealth of foliage, the luxuriance of flower and fruit, which lies between this bald sketch of a plant, and the giant pine of California, towering to the dimensions of a cathedral spire, or the Indian fig, which covers acres with its profound shadow, and endures, while nations and empires come and go around its vast circumference! Or, turning to the other half of the world of life, picture to yourselves the great finner whale, hugest of beasts that live, or have lived, disporting his eighty or ninety feet of bone, muscle, and blubber, with easy roll, among waves in which the stoutest ship that ever left dockyard would founder hopelessly; and contrast him with the invisible animalculæ — mere gelatinous specks, multitudes of which, could, in fact, dance upon the point of a needle, with the same ease as the angels of the schoolmen could, in imagination."

Now, Protoplasm is, according to Prof. Huxley, the "single, physical basis of life, underlying all these diversities of vital existence." "It is the clay of the potter: which, bake it and paint it as he will, remains clay, separated by artifice, and not by nature, from the commonest brick or sun-dried clod.' Thus it becomes clear — to him — "that all living powers are cognate; and that all living, forms are fundamentally of one character.' He does allow that there are two kinds of Protoplasm. The *vegetable*, which can manufacture fresh protoplasm from mineral compounds, and *animal*, which must procure it ready-made, or at most can only make it from pre-existing protoplasm.

The term, Sarcode, has never been but little used, and now has entirely given place to the term Protoplasm.

The term Germinal Matter, proposed by Beale, he himself has supplanted by the word Bioplasm, a shorter and more elegant term, and the one which, I think, will be adopted by the scientific world, as best expressing the idea — living matter.

Adopting this, then, as its scientific name, we inquire, first, as to its origin.

Origin of Bioplasm. — Of the origin of bioplasm, or living matter, the oldest account we have, and, I believe, it will be found to be the most reliable of any, and the one nearest in accord with the most advanced sciences, is the Mosaic account, which gives the origin of the vegetable kingdom, in these words. "And God said, Let the earth bring forth grass, the herb yielding seed, and the fruit tree yielding fruit, after his kind, whose seed is in itself. And the earth brought forth grass, and herb yielding seed after his kind, and the tree yielding fruit, whose seed was in itself, after his kind."

And of the origin of the animal world, it says: And God said, "Let the earth bring forth the living creature after his kind, the cattle, and creeping thing, and beast of the earth, after his kind: and it was so.

"And God made the beast of the earth after his kind, and cattle after their kind, and everything that creepeth upon the earth after his kind."

And of the origin of man, or the human species, it says: "And the Lord God formed man of the dust of the ground, and breathed into his nostrils the breath of life; and man became a living soul."

In a word, a Superior Intelligence bespeaks it into existence. Breathes into it life and immortality. How simple! How grand!

Let us next see what the investigations of science have accomplished in its search after the origin of living matter.

Among the oldest scientific writers, we find only what seems to us now, with our better means of analysis, rude attempts at defining the composition and origin of living matter; indeed, we may say, no analysis at all. In the Second century before Christ, Aristotle writes of the souls of living beings, and would teach us that there is something in living matter which does not belong to lifeless matter. But though the son of a physician, his writings pertain quite as much to to the metaphysical as the physical. From Galen, of the Second century after Christ, to Fallopius, of the Sixteenth century, the similar parts, or, as we should say, the simple parts, of the human body, are, the bones, cartilages, nerves, veins, arteries, and the like; in a word, what we now call the tissues. And all are pervaded by a peculiar force, the vital; but of the nature of this force, or the origin of these tissues nothing is told us.

But early in the Seventeenth century, the investigations into the constitution of the tissues advanced; for just at the close of the Sixteenth century, Jansens, of Holland, had invented the compound microscope (in 1590).

With it, Borelius, of Pisa, finds the pus corpuscles, and describes them as the animalculæ causing disease, and says he has seen them delivering their eggs. Boerhaave sees the blood corpuscles. Malpighi, the circulation of the blood in the lung of the frog. Harvey had discovered the circulation of the blood in 1616, but never saw it; Malpighi saw it in 1661. Leeuwenhoek had described the blood corpuscles in man and the lower animals, and examined most of the tissues, and Malpighi had described quite accurately the structure of plants. This was the work accomplished the first century with the microscope — the Seventeenth.

About the middle of the next (1757), Haller published his Elements of Physiology, and in this we find the first attempt at a rational development of the tissues. According to Haller, all the tissues are composed of fiber and a solid concrete. But of the origin of these, he does not tell us; though he does tell us that the fiber "is invisible, and can be distinguished only by the mind's eye."

Following next are the observations of Wolf, who finds all the tissues composed of globules, but does not pretend to tell how these globules originated.

The fiber, and then the globules, as ultimate elements of the tissues, are the results of the labors of the Eigtheenth century.

The next step, early in the Nineteenth century, was the raising of the cell to the place of first importance — a position it has held ever since.

This was accomplished chiefly by the labors of two men — Schleiden in the vegetable kingdom, and Schwann in the animal. But of the origin of the cells we get no new ideas. The globules of Wolf form the fibers of Haller, and the cells of Schleiden and Schwann. But how originated the globules? We get no reply, only that they arise out of a clear fluid.

And this is the essence of the theory of Spontaneous Generation. The history of the discussions relative to this subject, spontaneous generation, would form an interesting topic for an hour's discourse but we can give it now but a passing glance, and if you would study it further, I would refer you to an interesting monograph, recently published by Prof. Dalton. The earliest traces of the doctrine we find in the writings of Aristotle, two centuries before Christ. According to him, all animals produce either living young (are viviparous) or eggs from which the young are hatched (oviparous), or they produce larvæ, from which the fully developed insects are derived. And beside these, but considered as exceptional cases, were quite a class of animals, said to have been produced spontaneously from the mud-slime, in which they are found, or, as Huxley would say, by the combination of the natural forces inherent in the mud.

Observe, it is only the obscure classes, and those difficult to investigate, which are supposed to be of spontaneous origin. The shell fish was supposed to be produced in this way — such as clams, oysters, barnacles, and the like. So were also the sea-nettles, and sponges. Moths were produced spontaneously by the woollen fabrics in which they were found; so, too, maggots by the putrefying meat.

It was a favorite idea with Aristotle, that the life of the higher animal, on its death, passed by division, as it were, into the maggots, and other organisms which seemed to succeed them. And this is an idea which we find often revived by the supporters of the theory of spontaneous generation, and always concerning some obscure species or genus, about which we know so little, that it is difficult to prove that the contrary is true. As science advanced, the genealogy of the oyster and clam, is cleared up; the parent of the maggot is found in the fly, and next, only the entozoa, such as the tape worm and the pork worm, are claimed by the advocates of this theory, and these, in turn, having been proved to have come from a pre-existing animal, all naturalists were ready to subscribe to the statement of Cuvier in 1828.

Alluding to the statement of Pliny that some kinds of shell fish are produced in sandy deposits, by the spontaneous operation of nature, he says, "It is altogether untrue that animals are ever formed by spontaneous production. We are now perfectly acquainted with the eggs of these shell-fish and their whole mode of generation. The Aphya (the fish thought by the ancients to originate from the foam of the sea) is simply the young fry of larger fish ; and so of all the rest." But no sooner is the question settled concerning these animals, than its advocates find a new and exceedingly fertile field among the Infusoria which were just being discovered and studied with the compound microscope.

Needham, of England, a priest and naturalist of note, believed that infusoria would be developed, in organic solutions, under circumstances which precluded the possibility of the pre-existence of germs ; for example : He enclosed the juices of meat, hot from the fire, in glass vials, together with air, also previously heated, and shut out the atmosphere by "closely fitting corks." The infusoria and their germs are supposed to have been all destroyed, and no new ones can enter, as the bottles are "tightly corked." But infusoria do appear after a time, and he supposes they are produced by a kind of reorganization of the decaying organic materials.

But these opinions were vigorously combatted by Spallanzani, an Italian naturalist, who performed these same experiments with greater care. He hermetically sealed the glass flasks in which the organic infusions were placed, by melting their narrow necks. He next boiled the infusions for an hour, and then exposed them to such ordinary temperatures as were favorable to the development of infusoria.

The result of these experiments was that when the flasks were opened, not one of them showed the least appearance of animalculæ.

Schultze and Schwann, in Germany, still more carefully conducted these experiments, and with the same result.

And still later, Prof. Jeffries Wyman, of Harvard University, has shown the remarkable fact, that some of the infusorial germs are not destroyed by a temperature of 212° F., unless continued three or four hours; and recently Bastian has shown that they will resist a temperature of about 300° F., about the temperature at which sulphur melts. Now if many of these germs will withstand a temperature of from 212° to 300° F., as shown by the experiments of Wyman and Bastian, and if all the living beings from the higher infusoria to the highest animal — man — are *positively known* to be developed from preexisting living beings, is it not more reasonable to suppose that the germs of a few of the lowest infusoria are capable of withstanding a temperature of 350° F., or even higher, rather than to assert that they are spontaneously produced.

May it not be possible that some of the germs were in a portion of the apparatus not so highly heated as the rest, and so they escaped ?

Does not the term spontaneous generation simply mean, we do not know how they are produced? Has not this been proved to be its meaning in the past, and why may not such be the result now?

And is not the real object of the advocates of this theory to find a starting point for a method of development *without a Creator?* How unstable a foundation; how unworthy the name science! With every advance of knowledge, it must move on, to the confines of ignorance.

This brings us in our review to our own times. And what shall we say are the opinions of scientists now as to the origin of Bioplasm, or living matter? They may, I think, be conveniently classed as belonging to two schools—the materialistic and the vitalistic. The opinion of the materialists we will gather from Huxley; and we will give them as far as possible in his own words, to convey if we can thereby his exact meaning and no other.

Having analyzed *dead protoplasm*—I say dead protoplasm, for he could analyze no other — he must destroy life in order to a chemical analysis — having analyzed dead protoplasm and found it to consist of the simple chemical elements, carbon, hydrogen, oxygen and nitrogen, and existing in such proportions as to form carbonic acid, water and ammonia, he then asserts that "these compounds, like the elementary bodies of which they are composed, are lifeless. But when they are brought together, under certain conditions, they give rise to the still more complex body, protoplasm, and this protoplasm exhibits the phenomena of life."

We call different kinds of matter carbon, oxygen, hydrogen, and nitrogen, and speak of the various powers and activities of these substances as the properties of the matter of which they are composed.

"Mingle oxygen and hydrogen in proper proportions, and pass the electric spark through them and water is the result. Oxygen and hydrogen at 32° F., and far below, are gases, and their particles tend to rush away from one another with great force. But water, at the same temperature, is a strong, though brittle solid, whose particles tend to cohere in definite geometrical shapes."

"We do not assume that something called 'aquosity' has entered this water and made these changes which we see."

"Is the case in any way different when carbonic acid, water and ammonia disappear, and in their place, *under the influence of pre-existing living protoplasm*, an equivalent weight of the matter of life makes its appearance?" Yes, Mr. Huxley, but what is this "influence of pre-existing living matter?" May it not be just possible that this developes the protoplasm which is afterwards found? He replies, very humbly, "this influence of pre-existing living matter is something quite unintelligible."

But Prof. Owen goes even further than Huxley. He asserts that the formation of living beings out of inanimate matter, by the conversion of physical and chemical into vital modes of force, is going on daily and hourly.

In a word, living matter differs from other matter, only in possessing a peculiar combination of physical forces; a combination which may at any time occur by accident, or be invented by human ingenuity; and the chemist is approaching this grand combination, say they; he has already made in his laboratory urea and allantoin, with lactic acid, butyric oxalic and other organic acids—and the next step will be living matter itself. If so, it will be a long stride, in my opinion.

Undoubtedly the simple chemical elements oxygen, hydrogen, nitrogen, and carbon are present in all living matter, and at times, other elements which are of great importance to it in the performance of its functions; but to expect, by the study of the laws which regulate the combinations of these elements, to learn the nature or the functions of living matter, is as absurd as to attempt to study the architecture of Chicago before the great fire by the chemical analysis of its ashes. You might indeed find most of the chemical elements which composed its business houses, but how much could you learn by such an analysis of the business energy, or the financial standing of the firms they sheltered.

As well might you attempt to learn the speed of a horse by getting a chemist to analyze his muscles. Understand me, I am not disparaging chemical analysis, but attempting to show the absurdity of deciding questions relating to living matter by it.

But what shall we say of the vitalists? They believe there is a peculiar force or principle in organic matter, by which organized beings are enabled to take up the materials around them, and interchange them with their own, accomplishing thereby certain functions.

Whether this principle or spiritual agency is alike in the animal and vegetable kingdoms, or alike in the different kinds of animals and plants, are questions we cannot yet answer.

This activity is, as a famous zoologist Agassiz, I think, has said, "an invisible immaterial, superintending principle."

Whether this principle is related to, or can be correlated with the physical forces, such as gravity, light, heat, electricity, etc., that is, can be transformed into these, is a question we are not yet able to answer satisfactorily, though we incline to the negative.

If correlative, why are not these forces often seen to be transformed into the vital. It may be replied this is constantly occurring in the lowest orders of life, among the animalculæ and fungi. But why have the higher forms of living matter never appeared?

Again, if the vital force is correlated with heat, electricity, etc., when death occurs in part, or the whole of an organism, these forces should reappear with renewed power. But we know that the contrary is true—where there is increased vital activity, there is increased heat; and so too of the other forces we call physical, so far as they appear in the living organism at all. Witness the greatly increased heat of inflammation and the fevers. Instead, then, of the vital force being the resultant of the combined physical forces, it seems rather to liberate them and allow them to form new combinations or appear in new forms.

As to the origin of bioplasm, the vitalists reply briefly—from pre-existing bioplasm. The German school, with Virchow at their head, express it by saying every cell came from a pre-existing cell; and the English school, with Beale at its head, says every living particle came from a pre-existing living particle. But of the origin of the first cell or the first living particle science gives no satisfactory account, and revelation gives but an indistinct picture, for its object is not to teach science though it should not be at variance with truth in any department.

Bioplasm or living matter under the highest powers of the microscope at present in use, appears to consist of exceedingly small particles or globular masses, which are colorless, structureless and active.

The highest powers now used magnify an object 5000 diameters; so that if an object one foot in diameter could all be seen at once, it would appear to be a mile in length; a carpenter's two foot rule would appear to be an unwieldy measure of nearly two miles in length, which, as you know, would nearly reach from this building to the Court House square. And yet with an instrument of such a power as this, it is probable that only the larger masses of living matter can be seen and many are even too small to be seen at all.

*Growth of Bioplasm.*—Growth or development is one of the characteristics of bioplasm. Assimilation or the taking up of food or pabulum is a constant peculiarity of living matter, taking of the non-living and transforming it into the living, so that if there was no transformation of the bioplasm into tissues, there must be a constant increase of the living matter.

But *how* it grows or *how* the lifeless particles are transformed into the living, no microscopist has been able to see, and no chemist to determine by his analysis. This is the mystery of life.

*Function of Bioplasm.*—The function of bioplasm is to form tissues. Probably no mass of living matter, however small, is without its tissue envelope, which may be simply to protect, as in the epithelial cell, or besides this, may have a higher purpose, as in the muscle, to contract—the nerve to convey intelligence. Or the tissue at first formed may be transformed into still another substance—take as an example the bioplasm of the milk cell, whose tissue is transformed into water, sugar, casein, and the other ingredients of the milk. So, too, the tissue of the liver cell into the constituents of the bile; and so of the rest. The function of the bioplasm is to form all the tissues, and just here is a profound mystery.

Take for example one of the higher animals; the bioplasm of the adult has been all derived from the small mass in the germ. Each anatomical element of each and all of the tissues has its bioplasm, and each forms its own appropriate tissue with unerring certainty; and as yet we can determine no difference in the living matter from these different localities—nay, I might go further and with Huxley say we can detect no difference between the bioplasm of the animal and the vegetable; and how varied are the tissues formed. How unlike — the horny nail and the brain cell—the giant oak and the liver cell. How dissimilar the milk, the urine and the bile.

Remember I do not assert there is *no difference* in the bioplasm from these varied sources, but only that we are, as yet, unable to *detect* any, and that in each it is all derived from the single mass in the germ. And it is just here that the departments of pathology and therapeutics touch our subject.

The multiplication of bioplasm in the tissues may be so rapid that only an imperfect soft tissue is formed, and we have pus; or, though too rapidly growing, a firmer tissue may be developed and we have cancer, tubercle and the like; or this rapid growth may be general and we call it fever. Or, instead of the tissue once formed doing its appropriate work, it is transformed into some other substance, as in fatty degeneration of muscle. And, in your materia medica, what drugs will restrain or prevent the development of bioplasm? Will alcohol; will acids; will quinine? Are these then useful in fevers?

If externally, a solution of carbolic acid or permang. potass. will prevent the growth of bioplasm, will not these prevent suppuration?

But I must not follow these thoughts longer. They will be your topics of study during the coming winter—during your college years—yes, and during your whole lives as physicians. And now, gentlemen of the Entering Class, I invite you especially to the department of physiology and histology, as dealing most directly with these grand themes of living matter and the tissues, though the other departments will all treat of these more or less directly, as we have seen. I invite you to all of these departments of study. I invite you—especially those of you who are older in medical studies—to the hospitals and dispensaries. We throw open wide our doors and welcome you to work—to hard work—and we promise you on our part a corps of faithful instructors.

Intoxication in France.—M. Bergeron recently presented a lengthy paper to the Academy of Medicine of Paris on the subject of the terrible increase of intoxication in France from the use of distilled liquors. A meeting of the Academy, for the purpose of discussing the paper, was held early in October, and efforts are being made to secure the passage of laws which shall, by the imposition of fines upon those found intoxicated in the street, and by other means, remedy the present condition of society in this regard.—*Med. Rec.*

Duration of Pregnancy.—It is well known that since the days of Hippocrates the normal duration of pregnancy in the human female has been taken on an average to comprise ten lunar months, or ten menstrual periods of twenty-eight days. In some single cases the accurate reckoning of the duration of pregnancy is made impossible by the impossibility or uncertainty of fixing the day of conception. Notwithstanding these difficulties and sources of error in the calculation of the lenghth of pregnancy, there are still some exact observations to show that conceptions exist from the known average period of pregnancy, apparently connected with the individual peculiarities of the mother. In the lower animals, at any rate, these anomalies are the more numerous, in proportion as their average duration of pregnancy. For the human female Cederschjold first made the observation that the period of pregnancy lasts ten menstrual epochs, and Schuster and Berthold spoke in the same way. The author of a paper, Dr. Lowenhardt. in the *Arch. f. Gynakologie* remarks that we must remember that the menstrual period in the same woman does not always exhibit a normal and constant type; but he believes that he has found an approximately accurate means of calculation by observing the length of the last menstrual period, so that the distance of the commencement of the menstrual period before the last one to the last is to be taken as the tenth part of the length of the pregnancy. In order to shut out some errors, he reckons the length of the pregnancy in cases where the last observed interval is 26 days, at 256 to 265 days; in cases where the interval was 27 days, he calculates the duration of pregnancy at from 266 to 275 days; when the interval was 28 days, he calculates on a 276 to 285 days' pregnancy; in an interval of 29 days he reckons a pregnancy of 286 to 295 days; and from a 30 days' interval he calculates the pregnancy at 296 to 305 days. Since the interval of menstruation in the majority of women is 28 days, pregnancy must be estimated as a rule between 276 and 288 days.—*The Doctor.*

Nocturnal Incontinence Treated by Chloral.—Dr. Girolamo Leonardi (*L'Ippocratico*, 10th July), has found chloral useful even in adults long affected with nocturnal incontinence of urine, in the dose of one gramme dissolved in sixty grammes of water at bed-time. The patient is not to drink much at supper. He mentions one case of rapid cure in a young scrofulous male, aged 24, and another in a boy, aged 7. The cure was without delay.—*Ibid.*

